# Diversity of Molluscan Assemblage in Relation to Biotic and Abiotic Variables in Brown Algal Forests

**DOI:** 10.3390/plants11162131

**Published:** 2022-08-16

**Authors:** Martina Orlando-Bonaca, Domen Trkov, Katja Klun, Valentina Pitacco

**Affiliations:** Marine Biology Station Piran, National Institute of Biology, Fornače 41, SI-6330 Piran, Slovenia

**Keywords:** canopy-forming algae, coverage, molluscan assemblage, diversity, abiotic factors, Adriatic Sea

## Abstract

Canopy-forming macroalgae, mainly those belonging to the order Fucales, form the so-called brown algal forests, which are among the most productive assemblages in shallow coastal zones. Their vertical, branching canopies increase nearshore primary production, provide nursery areas for juvenile fish, and sustain understory assemblages of smaller algae and both sessile and vagile fauna. The majority of benthic invertebrates inhabiting these forests have larval stages that spend some time floating freely or swimming in the plankton. Therefore, canopy-forming macroalgae play an important role as species collectors related to larval supply and hydrodynamic processes. During the past several decades, brown algal forests have significantly reduced their extension and coverage in the Mediterranean basin, due to multiple interacting natural and anthropogenic pressures, with negative consequences also for the related fauna. The aim of this research was to examine how differences in macrophyte abundance and structure, as well as environmental variables, affect the associated molluscan communities in the shallow northern Adriatic Sea. Sampling sites with well-developed vegetation cover dominated by different canopy-forming species were selected in the shallow infralittoral belt of the northern Adriatic Sea in the spring–summer period of the years 2019 and 2020. Our results confirm the importance of algal forests for molluscan assemblage, with a total of 68 taxa of molluscs found associated with macrophytes. Gastropods showed the highest richness and abundance, followed by bivalves. Mollusc richness and diversity (in terms of biotic indices) were not related with the degree of development of canopy-forming species (in terms of total cover and total volume), nor with the ecological status of benthic macroalgae at different depths. On the contrary, the variability in molluscan taxa abundances was explained by some environmental variables, such as temperature, pH, light, and nitrates concentration.

## 1. Introduction

Canopy-forming species (mostly kelps and fucoids) are the worldwide dominant macroalgae in healthy shallow rocky temperate areas [1,2,3,4,5]. They are considered as autogenic ecosystem engineers, forming the so-called brown algal forests [6,7,8]. In recent decades, multiple anthropogenic pressures, such as coastal urbanization, eutrophication, increasing sediment loads, damage by fishing nets, and climate change, are directly or indirectly responsible for the worldwide decline of such habitats [7,9,10,11,12,13,14,15,16]. As a consequence of these interplaying pressures, canopy-forming species are being gradually replaced in many coastal areas by turf-forming smaller algae or even by barren grounds [17,18,19,20]. Some changes in macroalgal communities are also attributed to the synergy between anthropogenic and natural environmental factors, such as overgrazing by sea urchins [21,22,23,24] and herbivorous fish [25,26], and since fucoids are long-living algae that follow a long-term periodicity, their disappearance from coastal rocky bottoms is underlying local environmental degradation and habitat loss [27,28,29,30,31,32,33,34]. 

Mediterranean shallow rocky reefs, especially *Cystoseira sensu lato* spp. [35], are forming dense so-called brown algal forests, which are considered amongst the most productive assemblages in the shallow coastal area [36,37]. According to the European Red List of Habitats, photophilic communities with canopy-forming algae in Mediterranean infralittoral and upper circalittoral rock (code A3.13) are listed as endangered [38]. The complex habitats formed by *Cystoseira s.l.* spp. are nowadays locally disappearing at a shocking rate [13,39,40], and controlled experiments have confirmed the shift towards dominance by algal turfs, when brown algal canopies are severely damaged or removed [41]. This variation in the benthic structural complexity may in turn affect functions associated with forested rocky reefs, as the vertical, branching canopies of *Cystoseira s.l.* spp. increase coastal primary production [42,43], maintain assemblages of smaller algae, sessile and vagile invertebrates in the understory [44,45,46], and provide nursery areas for fish juveniles [47]. Therefore, canopy-forming macroalgae play an important role as species collectors related to larval supply and hydrodynamic processes [48]. In addition, the high spatial heterogeneity of brown algal forests results in exceptional species richness and high density of coastal fish assemblages [49,50].

Among the invertebrate fauna inhabiting brown algal forests, molluscan assemblages have been extensively studied and have often been found to be among the most abundant and dominant taxa, as well as an important food source for higher trophic levels [51,52,53,54]. However, despite many decades of marine research in the northern Adriatic, very little is known about the molluscan fauna associated with algal vegetation in this area [44]. 

Benthic macroalgae have been considered as an appropriate biological element for the assessment of the ecological status (ES) of Slovenian marine waters since the first evaluation in 2006 [55], in accordance with the requirements of the Water Framework Directive (WFD [56]). For the implementation of the WFD, two main ecological quality indices are applied for macroalgal communities on Mediterranean rocky bottoms: the Spanish index CARLIT (cartography of littoral and upper-sublittoral rocky communities) conceived by Ballesteros et al. [57], and the Greek Ecological Evaluation Index continuous formula (EEI-c) developed by Orfanidis et al. [58,59]. Due to the peculiarities of the northern Adriatic basin (high resuspension rate of sediments, as reported by Orlando-Bonaca et al. [60]), the Greek index was selected during the intercalibration process for the Mediterranean Sea [61] as the most appropriate index to assess the ES of Slovenian coastal waters. Since 2007, infralittoral macroalgal communities have been part of the national surveillance monitoring programme under the WFD and also the Marine Strategy Framework Directive (MSFD [62]) [63]. Both Directives require EU Member States to implement monitoring programmes for the assessment of Good Ecological Status (GES) and Good Environmental Status (GEnS) at least every six years. 

Additionally, considering the changes reported for canopy-forming species in the infralittoral belt of the northern Adriatic [31,32,33,64], empirical studies on molluscan assemblages were urgently needed. Therefore, a study of the molluscan assemblage inhabiting different types of algal canopies was also planned as part of a research project related to the assessment of the status of Adriatic brown algal forests (ARRS, J1-1702). In this context, the overall objective of the study presented in this paper was to (1) identify changes in canopy-forming species presence and abundance; (2) acquire a qualitative and quantitative characterization of molluscan assemblages associated with canopy-forming species; (3) test whether diverse canopy-forming species (with differences also in the ES evaluation) can support different molluscan assemblages; and (4) verify the influence of some environmental variables on the distribution of macroalgae and related molluscan assemblages in the northern Adriatic Sea. The results obtained also contribute to support the importance of brown algal forests as preferred habitats for the recruitment of molluscan taxa. 

## 2. Materials and Methods

### 2.1. Study Area and Sampling Sites

The Gulf of Trieste is a shallow semi-enclosed bay in the northernmost part of the Adriatic and Mediterranean Seas. The gulf stretches from Cape Savudrija (Croatia) to Grado (Italy) and encompasses the entire Slovenian coastline, with an average depth of about 21 m. The area is known for the lowest winter temperatures (mostly below 10 °C) in the Mediterranean, and the prevailing winds, which blow mainly from the northeast in an offshore direction [65]. The average salinity is about 37, mainly influenced by freshwater inflow from the Soča (Isonzo) River [66]. Water circulation is mainly counterclockwise in the lower layer and clockwise in the surface layer [67]. 

The rocky substrate along the Slovenian coast (46.7 km) consists of mainly flysch layers of Eocene age, alternating between firm sandstone and soft marl [68]. In the past, the coastal area has been affected by various anthropogenic impacts such as construction, intensive fishing, sewage discharges, and mariculture [60]. However, according to the TRIX index, which is a combination of loads (dissolved inorganic nitrogen and total phosphorus) and impact indicators (chlorophyll *a* and oxygen absolute deviation from saturation), water quality conditions of Slovenian coastal waters were recently assessed as elevated [69]. The natural variability of physicochemical variables was presented by Mozetič et al. [70] for Slovenian marine waters, and by Cozzi et al. [71] and Urbini et al. [72] for the wider area of the northern Adriatic Sea. The variety of phosphorus sources in the northern Adriatic, as opposed to nitrogen derived primarily from rivers, suggests that changes in atmospheric pollution and wastewater treatment are rapidly altering the availability of this limiting nutrient, with significant implications for productivity in this coastal area [71]. 

**Figure 1:**
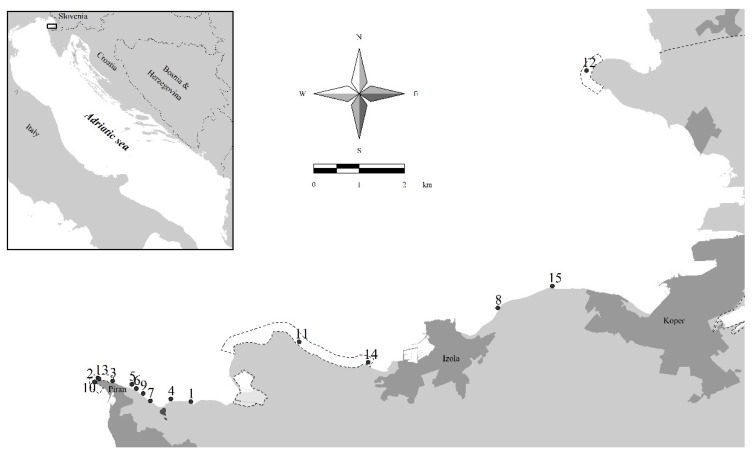
Sampling sites with canopy-forming macroalgae considered in the present study in Slovenian coastal waters (for details, see Table 1). The boundaries of three MPAs are outlined on the map (from left to right: Cape Madona Nature Monument near Piran, Strunjan Nature Reserve, and Debeli rtič Nature Monument). The three main coastal cities are Koper, Izola, and Piran.

Fifteen sampling sites with canopy-forming algal belts (Figure 1) were selected according to recent evaluations of their presence and abundance ([31,64]; authors’ unpublished data). Sites 11 and 14 are located in the Strunjan Nature Reserve, where the first reference site for macroalgae defined for Slovenian coastal waters is also present [55]. Sites 2, 3, 10, and 13 are located in the Cape Madona Nature Monument, where the second reference site for macroalgae is also placed [64]. Site 12 is located in the Debeli rtič Nature Monument, near the border with Italy. Sites 8 and 15 are located near a *Posidonia oceanica* (L.) Delile meadow, along the coastline between Koper and Izola [73]. All the other sampling sites are located from Piran toward the east, where the coastal belt is still in its natural state and, thus, is very important from a nature conservation point of view. 

### 2.2. Fieldwork and Laboratory Work

At selected sampling sites (Table 1), SCUBA visual surveys were performed at a depth of 1 m to max 12 m. In order to reduce the bias related to the sampling season, the sampling period was chosen to correspond with the moment of maximum development of *Cystoseira s.l.* species [64]. At each site, two horizontal transects [49], meter-marks of 50 m in length, were laid out at different depths, depending on the vegetation type on the bottom. Specifically, the transects were positioned at a constant depth where the canopy-forming algal belts were the most luxuriant. Usually, the first transect was laid out around 2 m depth, and the second around 4 m depth. Visual transects were conducted by a pair of SCUBA divers in order to recognize and count the fish (unpublished data). The data were collected within 1 m^2^, with a constant swimming speed, and a sample generally took 30 min. After that, three samples were randomly scratched off the rocky bottom (25 cm × 25 cm), collecting all macroalgae and associated sessile and vagile animals by hand, and placing them in plastic bags. The first and the second samples were collected on the two transects, while the third sample was scratched at the lower depth limit of the photophilic vegetation. In addition, seawater samples were collected in clean plastic bottles (prewashed with acid and Milliq) at all depths where benthic samples were collected, and water temperature, salinity, dissolved oxygen, and light intensity were also measured with underwater sensors (WTW Multi 3620 IDS and LI-COR Underwater Quantum Sensor Model Number: LI-192). All the measurements were taken between 10 and 12 a.m. All the samples were transported to the laboratory of the Marine Biology Station Piran of the National Institute of Biology for further analyses. 

All algal species were determined before fixation, and then each sample was preserved in 70% ethanol. Samples were sorted carefully, and the surface covered by each species (the orthogonal projection) was expressed in cm^2^ (4 cm^2^ = 1% coverage of sampling surface). Only species covering at least 1% of the sampling area were taken into consideration. In cases where it was impossible to measure coverage (as a measure of abundance) of morphologically similar taxa precisely, they were grouped together (as spp.). The “Algaebase” website [74] was used as an up-to-date source of nomenclatural information for macrophyte identification.

Samples were then sieved through a 0.5 mm mesh, and after a sorting process, molluscs considered to be alive at the time of sampling were determined to the lowest possible taxonomic level according to the most recent relevant literature and counted. Most juveniles were determined only to the family or genus level. The nomenclature follows WoRMS [75]. 

### 2.3. Physicochemical Parameters

The physicochemical variables considered are among the key environmental parameters measured in many studies dealings with algal communities, since they influence their growth and health [59,76].

**Nutrients:** Seawater samples for nutrient analysis (ammonium (NH_4_), nitrite (NO_2_), nitrate (NO_3_), phosphate (PO_4_), and silicate (SiO_4_)) were filtered through 0.45 µm filters (Millipore, MCE syringe filters) and immediately frozen at −20 °C until analysis. Nutrient concentrations were determined spectrophotometrically by segmented flow analysis (SFA) (autoanalyzer QuAAtro, Seal Analytical) according to methods described in Hansen [77]. The quality control is performed every year by participating in intercalibration programme and by obtaining good results (QUASIMEME Laboratory Performance Study).

**Dissolved organic carbon and total dissolved nitrogen:** Samples for dissolved organic carbon (DOC) and total dissolved nitrogen (TDN) analyses were filtered through pre-combusted GF/F (Whatman) filters through glass filtration system (pre-combusted at 500 °C for 4 h). DOC analyses were performed by a high-temperature catalytic method using a Shimadzu TOC-L analyzer. The calibration for non-purgeable organic carbon (NPOC) was performed with potassium phthalate and for TDN, potassium nitrate was used. The results were validated with Surface Sea Reference (SSR) water material for DOC (CRM Program, Hansell Lab). The reproducibility was lower than 2%.

**Total alkalinity and pH:** pH was measured within 1 h after sampling in the laboratory with pH meter (Metrohm 744) with Pt electrode. The in situ pH was recalculated with the following equation [78]:

*pHs* = pHm − α(T − Tm) (1)

where pHs: in situ pH; pHm: measured pH; α: constant (depending on in situ salinity and temperature measurements); T: in situ temperature; Tm: measured temperature in the laboratory.

Total alkalinity (*At*) was measured after first measuring the sample’s pH and then adding 15 mL of 0.01 M HCl to 50 mL of the sample. The pH was measured again after acid addition. The TA was calculated with the following equation [79]:

*At* = 4.0 − 1400 *aH/f* (2)

where *aH* = 10^−pH^; *f*: constant (depending on in situ salinity and temperature measurements).

### 2.4. Data Analysis

For the evaluation of the ES of macroalgal samples, the Ecological Evaluation Index continuous formula [59] was applied, which is a multimetric-scale-based biotic index that discloses the response of benthic macrophytes to anthropogenic pressures. This methodology comprises the separation of macrophyte taxa into two ecological state groups (ESG). ESG I comprises thick perennial (IA), thick plastic (IB), and shade-adapted plastic (IC) coastal water species, and angiosperm plastic (IA), thick plastic (IB), and shade-adapted plastic (IC) transitional water species. ESG II contains fleshy opportunistic (IIB) and filamentous sheet-like opportunistic (IIA) species, both in transitional and coastal waters [59]. The EEI-c value was calculated according to the equation of Orfanidis et al. [59]:

EEI-c = 0.4680 + 1.2088 × (x/100) + (−0.3583) × (x/100)^2^ + (−1.1289) × (y/100) + 0.5129 × (y/100)^2^ + (−0.1869) × (x/100) × (y/100) (3)

where x is the % coverage of taxa from ESG I, calculated as = (ESG IA*1) + (ESG IB*0.8) + (ESG IC*0.6), and y is the % coverage of taxa from ESG II, calculated as = (ESG IIA*0.8) + (ESG IIB*1).

In Slovenian coastal waters, the EEI-c has been previously applied to samples collected in the upper-infralittoral belt, at a depth range from 2 to 4 m [31,55,64]. However, for the present study, the index was applied also to samples collected in the lower infralittoral belt.

With respect to the molluscan community, the following univariate indices were calculated for each sample: the number of species (S_mol_), the number of individuals (N_mol_), the Shannon and Wiener diversity index (H’), and the Pielou index of equitability (J’) [80].

To test against a variation of biotic indices among sampling sites, a chi-square test applied to Kruskal–Wallis (KW) ranks [81] was run. 

The nonparametric Spearman rank-order coefficient (r_s_) [82] was used to test whether there was a correlation between macroalgal community parameters (EGSI, EGSII, EEI-c, algal cov, algal vol, N_cysto_, N_canopy_) and associated molluscan community indices (S_mol_, N_mol_, H’, J’).

To test the significance of multivariate differences in macroalgal composition among sampling sites and depths and molluscan composition among sampling depths and ES evaluated with EEI-c, a two-way permutational multivariate analysis of variance, PERMANOVA [83], was carried out on the three matrices: “macroalgal taxa coverage by sample”, “molluscan taxa abundance by sample”, and “molluscan feeding modes abundance by sample”. In order to reduce the weight of dominant species, “macroalgal taxa coverage by sample” matrix data were transformed using square root, whereas, given the low abundances of the dominant species, no pretreatment was needed for the “molluscan taxa abundance by sample” and “molluscan feeding modes abundance by sample” matrices. In all cases, Bray–Curtis similarity was used as the resemblance measure. A two-factorial design and the “permutation of residuals under a reduced model” with 9999 permutations were applied. For macroalgal composition, “site” with 15 levels and “transect” with 3 levels (T1 = about 2 m, T2 = about 4 m, T3 = lower limit of macroalgal community) were used; for molluscan composition and feeding modes, “transect” with 3 levels, and “ES” with 4 levels (“high”, “good”, “moderate”, and “bad”) were used. Pairwise comparison was also performed. These calculations were performed using the software package PRIMER v7 + PERMANOVA [83,84].

An RDA analysis was used to investigate the relationship between environmental variables and the coverage of macroalgal taxa, while a CCA analysis was used to examine the relationship between environmental variables and molluscan taxa. According to Zuur et al. [85], RDA is more suitable for communities with high alpha and low beta diversity, such as the macroalgal community in our case, while CCA is more suitable for communities with low alpha and higher beta diversity, such as the molluscan community of the present study. In both cases, only sites with a complete dataset for both abiotic and biotic variables were considered, and data were log-transformed. Molluscan taxa with frequency higher than 5% and macroalgal taxa with a total coverage considering the sum of all samples higher than 15% were displayed. Environmental variables were selected in order to reduce collinearity, as detected by the variance inflation factor (VIF) according to Zuur et al. [86], but also to obtain the best representation. A *p*-value < 0.05 was again chosen as the significance threshold. Analyses were performed using the vegan package [87] for R 4.0.2 [88].

## 3. Results

### 3.1. Ecological Status Evaluation of Macroalgal Communities 

The ES achieved by macroalgae at 15 sampling sites was assessed as high for almost all the samples collected at around 2 m depth, with the exception for sampling site 4 (Table 2). Additionally, the ES for samples collected around 4–5 m of depth was still high or good, with the exception for sampling site 10. At stations 5, 7, 10, and 12, samples collected at the lower depth limit of the photophilic vegetation were evaluated as moderate or, in one case, as bad (Table 2).

All the species from the genus *Cystoseira s.l.* and the red alga *Halopithys incurva* (Hudson) Batters were considered as canopy-forming species (both ESG I taxa). The number of canopy-forming species varied from 1 to 3 per sample, while the number of thalli stayed between 1 and 18 per sample. The coverage (%) of canopy-forming species was the highest in the samples at 2 m depth (Table 2), and in some samples (at stations 8 and 10) was even higher than 100%, due to different layers of canopies. The most frequent species was *Cystoseira compressa* (Esper) Gerloff & Nizamuddin (found in 23 of 44 samples), followed by *Gongolaria barbata* (Stackhouse) Kuntze 1891 (13 samples), *Cystoseira corniculata* (Turner) Zanardini (12 samples), *H. incurva* (10 samples), and *Ericaria crinita* (Duby) Molinari & Guiry and *Cystoseira foeniculacea* f. *latiramosa* (Ercegović) A. Gómez Garreta, M.C. Barceló, M.A. Ribera & J.R. Lluch (both in two samples). 

### 3.2. Molluscan and Algal Assemblages in Relation to Abiotic Factors

A total of 68 molluscan taxa (Supplementary Materials Supplement 1) were found associated with benthic vegetation dominated by canopy-forming species in the infralittoral belt of the northern Adriatic Sea. Gastropoda showed the highest richness with 42 taxa (representing 53% of the total mollusc abundance), followed by Bivalvia with 24 taxa (46% of total abundance), and Polyplacophora, with only two taxa (0.6% of total abundance). The taxa belonged to 34 families, of which Rissoidae, Muricidae, and Trochidae displayed the highest number of species (Supplementary Materials Supplement 1). The most frequent and abundant taxa were three gastropods: *Cerithium vulgatum* Bruguière, 1792 complex, *Jujubinus exasperatus* (Pennant, 1777), and *Tritia incrassata* (Strøm, 1768) (Supplementary Materials Supplements 1 and 2). All these taxa were present in both juvenile and adult form.

The identified molluscan taxa belonged to five feeding guilds: micrograzers, suspension feeders, deposit feeders, predators, and spongivores (Supplementary Materials Supplement 1). Suspension feeders were the dominant group both in terms of species richness (22 taxa, the majority from Mytilidae and Veneridae families) and abundance, followed by predators (21 taxa, the majority from the family Muricidae) and micrograzers (20 taxa, the majority from Trochidae and Rissoidae families). Spongivores and deposit feeders were minimally present, with four and one species, respectively (Supplementary Materials Supplement 1).

**Figure 2:**
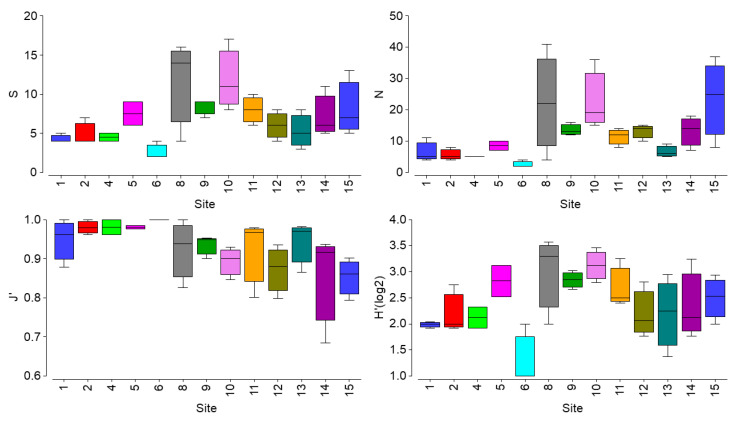
Boxplots showing values of indices for the molluscan community. S = molluscan richness, N = molluscan abundance, J’ = Pielaou index, H’(log2) = Shannon–Wiener diversity index.

No significant differences of biotic indices (Smol, Nmol, J’, and H’) were observed for the molluscan community among sampling sites (KW chi-squared, *p* > 0. 05, Figure 2). 

The majority of the parameters for macroalgal communities were correlated among each other. Specifically, ESGI, EEI-c, algal coverage, algal volume, number of Cystoseria s.l. species (N_cysto_), and number of canopy-forming species (N_canopy_) were positively correlated, whereas ESGII was negatively correlated with EEI-c, N_cysto_, and N_canopy_ (Table 3). The same was true for the indices for molluscs: S_mol_, N_mol,_ and H’ were positively correlated, while J’ was negatively correlated with S_mol_ and N_mol_ (Table 3). Conversely, no significant correlation was found between parameters for macroalgae and for molluscs (Table 3). 

Considering the resemblance matrix based on the Bray–Curtis similarity measure, no significant differences in macroalgal species composition were found among sites, or among samples at different depths (PERMANOVA, *p* > 0.05, Supplementary Materials Supplement 2). No differences in molluscan composition were observed among samples at different depths (PERMANOVA, *p* > 0.05, Supplementary Materials Supplement 3) nor among samples with different ES (PERMANOVA, *p* > 0.05, Supplementary Materials Supplement 2), and the pairwise comparison revealed no significant difference as well (PERMANOVA pairwise tests, all *p* > 0.05, Supplementary Materials Supplement 3). Considering the abundances of molluscan different feeding guilds, no significant difference was observed among samples at different depths (PERMANOVA, *p* > 0.05, Supplementary Materials Supplement 2), nor among samples with different ES (PERMANOVA pairwise tests, *p* > 0.05, Supplementary Materials Supplement 2), and the pairwise comparison revealed no significant difference as well (PERMANOVA pairwise tests, all *p* > 0.05, Supplementary Materials Supplement 2).

**Figure 3:**
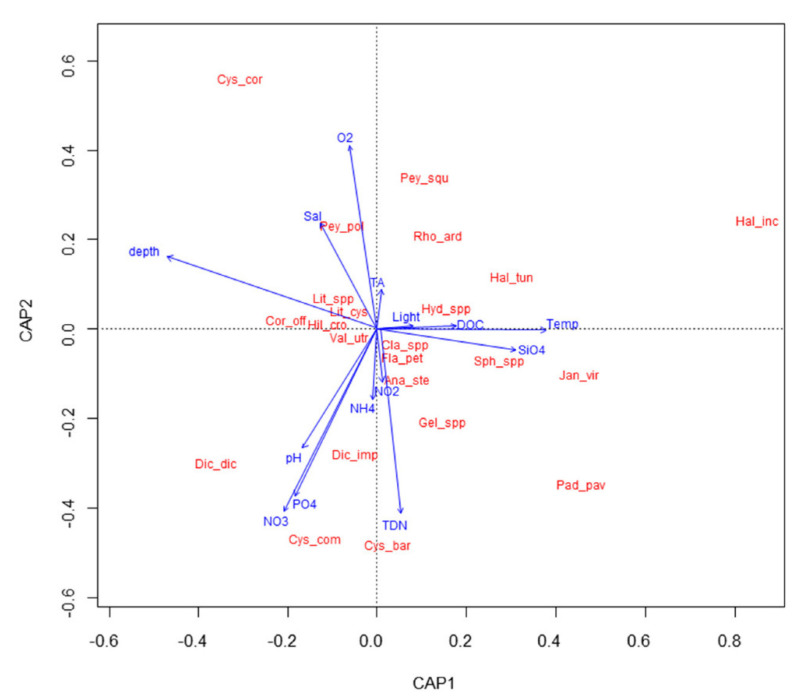
RDA biplot showing relationships among macroalgal taxa and environmental parameters. Total inertia: 3.647, eigenvalues for axis 1 = 0.751, eigenvalue for axis 2: 0.626. Cys_cor = *Cystoseira corniculata*; Cys_comv = *Cystoseira compressa*; Cys_bar = *Gongolaria barbata*; Hal_inc = *Halopithys incurva*; Pey_squ = *Peyssonnelia squamaria*; Pey_pol = *Peyssonnelia polymorpha;* Rho_ard = *Rhodymenia ardissonei*; Hal_tun = *Halimeda tuna*; Hyd_spp = *Hydrolithon* spp.; Dic_dic = *Dictyota dichotoma*; Dic_imp = *Dictyota implexa;* Pad_pav = *Padina pavonica*; Gel_spp = *Gelidium* spp.; Jan_vir = *Jania virgata*; Ana_ste = *Anadyomene stellata;* Cor_off = *Corallina officinalis*; Sph_spp = *Sphacelaria* spp.; Fla_pet = *Flabellia petiolata*; Cla_spp. = *Cladophora* spp.; Lit_spp. = *Lithophyllum* spp.; Lit_cys = *Lithophyllum cystoseirae*; Val_utr = *Valonia utricularis*; Hil_cro = *Hildenbrandia crouaniorum*.

With RDA, a diagram was obtained that shows the main pattern of variation in the macroalgal assemblage composition as accounted for by the environmental variables, and it also shows, in an approximate way, the distribution of the taxa along each environmental variable (Figure 3). Macroalgal communities are significantly affected by depth, temperature, SiO_4_, pH, NO_2_, oxygen, and salinity (RDA model, *p* < 0.05, Supplementary Materials Supplement 3). The RDA biplot shows a gradient of increasing water temperature and SiO_4_, and decreasing depth from left to right, and a gradient of increasing pH, NO_2_, and decreasing oxygen and salinity from top to bottom of the graph. Among *Cystoseira s.l.* species, *C. corniculata* shows a preference for deeper waters, with more oxygen but less nutrients and light intensity, while *G. barbata* and *C. compressa* are more abundant in shallower waters with more nutrients and higher pH.

**Figure 4:**
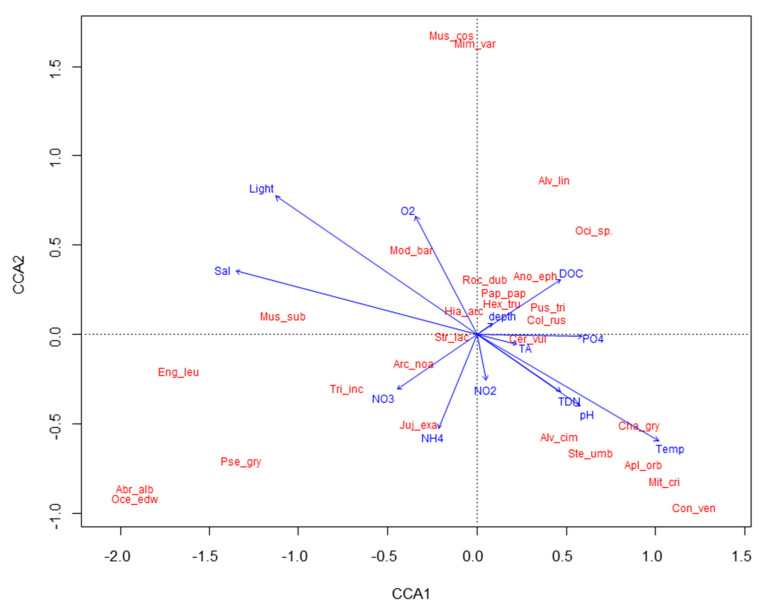
CCA biplot showing relationships among molluscan taxa and environmental parameters. Total inertia: 3.2217, eigenvalues for axis 1 = 0.414, eigenvalue for axis 2: 0.346. Mus_cos = *Musculus costulatus*; Mim_var = *Mimachlamys varia*; Alv_lin = *Alvania lineata*; Oci_sp. = *Ocinebra* sp.; Mod_bar = *Modiolus barbatus*; Mus_sub = *Musculus subpictus*; Roc_dub = *Rocellaria dubia*; *Ano_eph = Anomia ephippium*; Pap_pap = *Papillicardium papillosum*; Hex_tru = *Hexaplex trunculus*; Hia_arc = *Hiatella arctica*; Pus_tri = *Pusia* cf *tricolor*; Col_rus = *Columbella rustica*; Str_lac = *Striarca lactea*; Cer_vul = *Cerithium vulgatum* complex; Arc_noa = *Arca noae*; Eng_leu = *Enginella leucozona*; Tri_inc = *Tritia incrassata*; Juj_exa = *Jujubinus exasperates*; Cha_gry = *Chama gryphoides*; Alv_cim = *Alvania cimex*; Pse_gry = *Pseudochama gryphina*; Ste_umb = *Steromphala umbilicaris*; Apl_orb = *Aplus dorbignyi*; Mit_cri = *Mitrella* cf *scripta*; Con_ven = *Conus ventricosus*; Oce_edw = *Ocenebra edwardsii* complex; Abr_alb = *Abra alba*.

The CCA yielded a diagram that shows the main pattern of variation in the molluscan assemblage as accounted for by the abiotic variables and the distribution of single taxa along each variable (Figure 4). Molluscan assemblages are significantly affected by water temperature, pH, light, and NO_2_ (CCA model, *p* < 0.05, Supplementary Materials Supplement 3). The CCA plot shows a gradient of increasing water temperature and pH, and decreasing light from left to right, and a gradient of increasing NO_2_, from top to bottom of the graph. The majority of molluscan taxa exhibit a preference for lower light conditions and moderate nutrient levels. 

## 4. Discussion

### 4.1. Characterization of Algal and Molluscan Assemblages

The sampling sites selected for this research are mainly located in areas of low anthropogenic impact, where the coastline is still in an (almost) natural state [55,64]. Therefore, a high or good ES was expected according to the evaluation of macroalgae (Table 2), at least for the samples collected in the upper-infralittoral belt (which in Slovenian waters ranges from 1 m to about 4 m of depth), where canopy-forming species should predominate in rocky, healthy environments. 

*E. crinita* was found in only two samples collected in the upper-infralittoral belt (Table 2). It is known that in areas with some disturbance, algal communities dominated by *E. crinita* show a shift towards less structural complexity and homogenization of the habitat [89]. In addition, *Cystoseiretum crinitae* subass. *Halopithetosum incurvae* and *Cystoseiretum crinitae* subass. *Cystoseiretosum compressae* have been replaced by *Cystoseiretum barbatae* in some Slovenian coastal areas [31,55], and this shift is also confirmed by the results of this study. It is conceivable that multiple and cumulative stressors have caused the decline of *E. crinita* and other sensitive canopy-forming species. Even at such sites with high/good ecological values, canopy-forming species show different preferences for abiotic variables (Figure 3), with *G. barbata* and *C. compressa* tolerating low organic enrichment in shallower waters. Indeed, Cormaci and Furnari [90] previously reported that *C. compressa* dominates unperturbed sites with mild pollution, while Vukovič [91] concluded that *G. barbata* can grow at sites with higher sedimentation rates and low organic pollution. *H. incurva*, which in some cases was also found in the lower-infralittoral belt (see sampling site 6 in Table 2 and Figure 3), is also reported to adapt to unsteady biotopes [90]. *C. corniculata* was almost never found in very shallow waters, showing a preference for the lower-infralittoral belt (Table 2 and Figure 3), which was also reported for the Croatian coast of Istria [27]. Moreover, Devescovi [92] described that the species, similar to *G. barbata*, is sensitive to disturbances characteristic of harbor areas. However, surprisingly, *C. foeniculacea* f. *latiramosa* was found during the present research [93], after being absent from Slovenian marine waters for more than four decades and having been found only once before [94]. The species is considered rare and endangered, and it has already disappeared from some areas of the Mediterranean Sea [13].

The moderate (and in one case bad) ES was assessed for some samples with extremely low coverage of *Cystoseira s.l.* species and *H. incurva* (Table 2), or high coverage of opportunistic species, indicating some degree of local pressure. Since *Cystoseira s.l.* spp. are characterized by limited propagule dispersal and slow growth, they are unable to rapidly respond to anthropogenic impacts [95]. In the Slovenian coastal area, anchoring in the upper-infralittoral belt remains a problem (pers. observ.), as well as poor light conditions due to high sediment resuspension [60]. Fishing with trawl nets can be excluded due to the very shallow rocky bottom where canopy-forming species grow, as well as nutrient enrichment and chemical pollution, since a substantial decrease of the eutrophication has been reported for the northern Adriatic [70,96], confirming a reduction in phosphate and ammonia levels [97]. The results of the analyses of physicochemical parameters in our study are consistent with this previous research. However, land–sea interaction due to runoff of pesticides and other chemicals has also been implicated as a cause of *Cystoseira s.l.* spp. decline [98]. Moreover, the grazing effect of native herbivorous species was very recently reported to be one of the main pressures on young *Cystoseira s.l.* thalli [32,33]. *Sarpa salpa* (Linnaeus, 1758), which is able to drastically reduce algal and seagrass canopies, has especially been defined as an important ecosystem modifier, as the smallest individuals (forming smaller shoals) have been found to feed primarily on macroalgae, while the largest individuals (grouped in larger shoals) feed exclusively on seagrasses [99]. Eventually, increasing winter temperatures in the area [15,33] and exceptional wind periods [32] can lead to serious biological anomalies and the loss of the reproductive potential of *G. barbata*. Orlando-Bonaca et al. [31] already reported a decline in the overall *Cystoseira s.l.* spp. coverage in the Slovenian coastal area, but currently it is not possible to compare the results of the assessment of the ES according macroalgae with the status of algal communities in the adjacent areas of the northern Adriatic Sea, since macroalgae are not sampled with the same methodology (EEI-c) along the Italian and Croatian coasts. However, Iveša [100] hypothesized for the nearest geographical area, the western Croatian coast of Istria, that high summer temperatures and benthic mucilage (microalgal and macroalgal blooms) play a crucial role in the decline of *Cystoseira s.l.* forests. These preliminary observations for the northern Adriatic Sea follow the reported effects of increasing temperatures on marine forests in other Mediterranean regions [7,8,101]. Climate change is globally causing unprecedented alterations in marine ecosystems and is known to induce species redistributions, i.e., under a warmer climate, species would move towards higher latitudes, higher altitudes, or deeper waters [102]. However, in the shallow northern Adriatic Sea these migrations are not possible, since the area is the northernmost part of the whole Mediterranean Sea.

Regarding the molluscan assemblage associated with canopy-forming species, the results of the present study confirm that Gastropoda, followed by Bivalvia and Polyplacophora, are the most numerous groups of molluscs in terms of species richness and abundance, as evidenced by Pitacco et al. [44] for the same geographical area. Molluscan communities in algal assemblages have been previously reported to be dominated by gastropods, with a smaller number of bivalves, and only a few chiton species [51,103,104,105,106,107,108,109,110].

Although it is relatively difficult to compare molluscan communities between different areas, our results are mainly consistent with similar studies conducted in other Mediterranean areas. Many authors have noted that the family Rissoidae is represented by the largest number of species in *Cystoseria* assemblages [44,53,54,108,109]. Among Bivalvia, *Modiolus barbatus* (Linnaeus, 1758) and *Musculus costulatus* (Risso, 1826) (see Supplementary Materials Supplement 1), have been previously recognized as the most abundant and frequent species in vegetated sites [44,51,103,104,106,107]. Among Gastropoda, *J. exasperatus* and *T. incrassata* were the most common and abundant species, which is also consistent with previous studies [44,51,103,104,106,107,111]. However, the high frequency of occurrence and abundance found for *C. vulgatum* complex (see Supplementary Materials Supplement 1) is somehow surprising for this area, compared to Pitacco et al. [44], while the abundance of *Bittium reticulatum* (da Costa, 1778) was relatively low compared to the same study. These results may indicate cyclic population patterns in molluscs, which are evident in the form of fluctuations and annual abundance patterns over periods of time [112]. Such cyclic patterns in molluscs are also known due to parasite loads [113] and are currently observed in the case of the largest Mediterranean bivalve *Pinna nobilis* Linnaeus, 1758, which is in severe decline [114,115]. However, the cyclical population patterns of certain species could also be related to other interplaying factors, such as a changing resource situation or a prey–predator relationship (for review, see [112]). 

Based on the feeding strategy, the most frequent and abundant species were suspension feeders, micrograzers, and predators, which is comparable with other studies on the molluscan communities associated with macroalgae in the Mediterranean Sea [44,54,108]. While suspension feeders feed on suspended particles and planktonic organisms from the water column, micrograzers feed mainly on epiphytic microalgae and diatom films, as well as sediment particles trapped by the branching canopy of macroalgae [116,117]. This indicates that canopy-forming algae serve primarily as habitat and substrata for larval settling rather than food for most molluscan species, which agrees with Chemello and Milazzo [51] and also supports the observations that phytofauna mostly do not feed on host plant tissues [118]. Sedimentation rate and turbidity (measured as solids in suspension) are known to be the most important factors affecting the epifauna associated with macroalgae, along with water movement, nutrient content, and plant morphology [119]. Suspended particles represent an important food source for suspension feeders, which were the dominant group in terms of species richness and abundance. This is not surprising considering that the mean value of total suspended sediment in the area is quite high (39.9 ± 15.8 g m^−1^) and consists of 40% organic material [120], which is known to be positively correlated with the assemblage of molluscs and macroalgae [106]. Sánchez-Moyano et al. [106] found that solids in suspension have a positive effect on detritivorous molluscan species, for which sediment retention on algae is an important food source. In addition, the importance of the sediment-holding capacity of a particular substrate is known to have a positive effect on the population density of many prosobranchs, particularly detritivores [121,122,123,124]. However, in the present work, strictly detritivorous molluscs were poorly represented, but it should be considered that many species of micrograzers also feed on sediment particles [116,117]. For the molluscan community, the most important aspect of the sediment is its quality, which goes beyond the percentage of containing organic matter and its granulometric composition [125]. 

Moreover, sediments that cover algae and provide food for detritivores also have negative effects on macroalgal growth [106,126]. Sedimentation thus has a positive effect on detritivores, while limiting macroalgal growth and reducing the available habitat for other molluscan species, as well as the detritivores themselves. The reduced available space on the plant affects the abundance of molluscs due to the reduction of microhabitat [127], but also due to the less available food (retained sediment and epiphyte flora) [128,129]. In addition, sedimentation can negatively impact benthic suspension feeders by potentially clogging bivalve siphons, causing stress, and being disturbed by sand [130,131,132].

The composition of molluscan community in terms of feeding modes did not differ significantly among depth, nor among sites with different ES.

### 4.2. Biotic and Abiotic Factors Affecting Molluscan Assemblages

The epifauna associated with macroalgae is influenced by the physical properties of the algal thalli and the physical medium [133]. Despite previous research [51,108,118,134,135] on the influence of algal morphological characteristics (algal architecture) on invertebrate assemblages, our results showed that in the northern Adriatic Sea, different algal variables (ESGI, EEI-c, algal coverage, algal volume, number of *Cystoseria s.l.* species, and total number of canopy-forming species) do not significantly influence molluscan assemblages. Although there is some evidence of a decline in the overall cover of canopy-forming species [31,64], it appears that the extent and abundance of macroalgal assemblages in the area are still sufficiently high for settlement of diverse molluscan larval stages. Thus, availability of vegetated habitats in the area is not currently a limiting factor for molluscan assemblages, and, consequently, the type of vegetated habitat does not strongly influence the diversity and abundance of molluscs, but there are other factors that determine the variability in molluscan communities. 

However, our results show significant effects of environmental parameters on molluscan communities, of which water temperature, pH, light, and nutrients had the greatest influence. With the exception of pH, the other environmental variables mentioned are closely related to the amount of available food. The presence, type, and availability of food is considered to be the most important factor affecting the distribution and abundance of different molluscan species on rocky bottoms [136]. In addition, the available food was recognized by Sánchez-Moyano et al. [106] as one of the most important factors determining molluscan communities associated with macroalgae.

The observed effect of nutrients on molluscan communities is also related to the availability of food, since moderate nutrient concentrations have a positive effect on algal growth [137,138]. Epiphytic algae have a significant impact on the molluscan community, providing food for one of the dominant trophic groups of micrograzers. Additionally, in our study, a moderate nutrient supply had a positive effect on habitat-forming macroalgae such as *G. barbata* and *C. compressa* (see Figure 3), which provide habitat and shelter for a diverse molluscan assemblage. However, it is also known that a more significant increase in nutrient concentrations leads to a decline in canopy-forming species [58,139], resulting in a loss of habitat for associated molluscan communities. 

Because molluscan taxa were not statistically significantly affected by water depth, we conclude that reduced light conditions positively affecting molluscan species are primarily correlated with particles in the water column due to sedimentation and resuspension of sediment [60,120], rather than concentrations of plankton [140]. 

The water temperature that had a significant effect on the molluscan taxa was mostly associated with the time (date) of sampling. Temperature [133,141] and seasonality [141] are known to have major effects on the structure and composition of benthic communities in temperate sea. Seasonal changes in molluscan communities are correlated with environmental variables and reproductive cycles [142]. It is known that high abundance and species richness of molluscs in spring and summer correlates with the biomass of algae and seagrasses [128,143], which both provide food and habitat for the molluscan community [144,145]. The maximum abundance of molluscs is synchronized with available food sources, leading to successful recruitment and consequently high abundance and species richness [146]. For example, *C. vulgatum*, the most frequent and abundant species in this study, is known to be most abundant in summer (August), which correlates with the high phosphate concentration of water temperature reflected in the greatest amount of available food and refuge [138]. On the other hand, a negative correlation has been observed between the morphological characteristics of canopy-forming macroalgae and seawater temperature [147], which consequently leads to a reduction in available habitat for molluscan assemblages.

Light, nutrients, and temperature have indirect effects on molluscan communities through available food and habitat [109,137,138], while pH has direct effects on them. The negative effects of decreasing pH on molluscan communities are likely related to the negative effects of acidic pH on calcification and reproduction in molluscs [148]. As a result, acidic pH is unfavorable for the occurrence of molluscs. It is also known that molluscs prefer slightly alkaline environments [149,150,151], which is also confirmed by Pennak [152], who observed a larger mollusc population in alkaline lakes compared to acidic lakes. One of the most probable reasons for the decrease in pH is the influx of freshwater [148], which is mainly related to the submerged freshwater springs, which are quite abundant in the area, as well as to the river tributaries in the northern Adriatic [153]. Furthermore, these results point to the burning problem of ocean acidification for molluscan communities [154].

The results of the present study show that different environmental parameters have different and interplaying effects on molluscan communities, affecting their species richness and abundance. Environmental parameters can have a positive effect on some mollusc species and a negative effect on others, as well as on macroalgae, on which molluscs depend [58,106,130,131,132,137,138,139,147]. Therefore, it is difficult to quantify the importance of single factors to molluscan communities [155,156]. In the context of anthropogenic impacts and climate change, future research on responses of canopy-forming species and associated molluscan assemblages should be planned, also to facilitate recently started restoration activities on canopy-forming species [32,33]. 

## Figures and Tables

**Table 1:** Sampling sites for macrophytes and molluscan fauna, sampling dates, and depths where the three samples were collected (with the exception of site 4, where only two samples were scratched).

Sampling Site	Lat N	Long E	Date	Sampling Depths (m)
1	45°31.571′	13°35.385′	19 July 2019	1.6; 3.7; 5.2
2	45°31.859′	13°33.816′	23 July 2019	2.0; 4.0; 8.8
3	45°31.824′	13°34.065′	26 July 2019	2.2; 4.6; 9.8
4	45°31.604′	13°35.049′	20 August 2019	2.4; 3.8
5	45°31.782′	13°34.391′	21 August 2019	2.8; 5.7; 12.0
6	45°31.732′	13°34.466′	29 August 2019	2.9; 5.3; 10.2
7	45°31.583′	13°34.700′	17 September 2019	3.0; 4.0; 8.0
8	45°32.658′	13°40.595′	1 July 2020	1.9; 2.2; 3.5
9	45°31.672′	13°34.580′	2 July 2020	2.5; 4.5; 8.7
10	45°31.812′	13°33.759′	6 July 2020	2.2; 4.4; 4.8
11	45°32.272′	13°37.226′	8 July 2020	2.2; 4.2; 8.0
12	45°35.478′	13°42.133′	9 July 2020	1.3; 3.0; 5.0
13	45°31.849′	13°33.837′	16 July 2020	2.5; 4.1; 8.2
14	45°32.022′	13°38.392′	20 July 2020	2.1; 4.2; 5.2
15	45°32.913′	13°41.523′	27 July 2020	2.0; 3.5; 4.3

**Table 2:** Evaluation of the ecological status achieved by macroalgae at 3 different depths at 15 sampling sites, number and names of canopy-forming species (CFS), and number and coverage (%) of thalli of CFS. Ecological status classes: high, in blue (10 ≥ EEI-c > 8.09), good, in green (8.09 ≥ EEI-c > 5.84), moderate, in yellow (5.84 ≥ EEI-c > 4.04), bad, in red (2.34 ≥ EEI-c).

Sampling Site	Sampling Depth (m)	EEI-c	N of CFS	Names of CFS	N of Thalli of CFS	Coverage (%) of CFS
1	1.6	9.97	1	*G. barbata*	7	53
	3.7	6.62	2	*G. barbata*, *C. compressa*	3	16
	5.2	6.42	1	*C. compressa*	2	7
2	2.0	10.00	2	*H. incurva*, *C. compressa*	12	53
	4.0	7.17	1	*C. corniculata*	1	8
	8.8	6.34	1	*C. compressa*	1	4
3	2.2	10.00	1	*G. barbata*	5	60
	4.6	9.45	3	*G. barbata*, *C. compressa*, *C. corniculata*	4	19
	9.8	9.55	1	*C. corniculata*	2	70
4	2.4	4.81	1	*H. incurva*	1	2
	3.8	5.93	0		0	0
5	2.8	9.89	2	*G. barbata*, *C. corniculata*	3	14
	5.7	8.63	3	*G. barbata*, *C. compressa*, *C. corniculata*	4	18
	12.0	5.28	1	*C. corniculata*	2	48
6	2.9	10.00	1	*G. barbata*	3	38
	5.3	10.00	2	*G. barbata*, *C. corniculata*	7	62
	10.2	9.12	2	*H. incurva*, *C. corniculata*	7	56
7	3.0	8.59	1	*G. barbata*	3	35
	4.0	6.21	1	*C. compressa*	5	5
	8.0	4.17	1	*C. compressa*	1	3
8	1.9	10.00	2	*H. incurva*, *C. compressa*	8	103
	2.2	10.00	3	*H. incurva*, *C. compressa, G. barbata*	11	101
	3.5	7.26	1	*C. compressa*	1	3
9	2.5	10.00	2	*G. barbata*, *C. compressa*	7	78
	4.5	9.80	2	*G. barbata*, *C. compressa*	8	52
	8.7	10.00	1	*C. corniculata*	3	75
10	2.2	10.00	2	*C. compressa, E. crinita*	17	116
	4.4	5.49	1	*C. compressa*	1	4
	4.8	2.00	1	*C. compressa*	2	6
11	2.2	10.00	1	*H. incurva*	4	70
	4.2	8.15	1	*C. compressa*	6	29
	8.0	8.17	2	*C. compressa, C. corniculata*	3	9
12	1.3	8.51	1	*H. incurva*	6	52
	3.0	8.72	1	*H. incurva*	1	4
	5.0	4.19	1	*C. compressa*	1	2
13	2.5	10.00	1	*C. compressa*	18	66
	4.1	10.00	2	*C. compressa, C. corniculata*	9	32
	8.2	7.52	1	*C. corniculata*	3	21
14	2.1	9.49	3	*G. barbata*, *C. compressa, E. crinita*	6	40
	4.2	10.00	1	*H. incurva*	6	75
	5.2	6.34	0		0	0
15	2.0	10.00	2	*H. incurva*, *C. compressa*	12	80
	3.5	7.55	1	*Cystoseira foeniculacea* f. *latiramosa*	1	2
	4.3	7.39	1	*Cystoseira foeniculacea* f. *latiramosa*	2	25

**Table 3:** Spearman coefficient (r_s_) between parameters for macroalgal communities (EGS I, EGS II, EEI-c, algal cov, algal vol, N_cysto_, N_canopy_) and for molluscan-associated assemblages (S_mol_, N_mol_, H’, J’).

		ESG I	ESG II	EEI-c	Algal cov	Algal vol	N Cysto	N Canopy	S_mol_	N_mol_	J’
ESG II	r_s_	−0.51									
	*p*	<0.05									
EEI-c	r_s_	0.94	−0.73								
	*p*	<0.05	<0.05								
Algal cov	r_s_	0.83	−0.99	0.67							
	*p*	<0.05	NS	<0.05							
Algal vol	r_s_	0.55	−0.25	0.51	0.66						
	*p*	<0.05	NS	<0.05	<0.05						
N Cysto	r_s_	0.45	−0.36	0.43	0.28	0.38					
	*p*	<0.05	<0.05	<0.05	NS	<0.05					
N canopy	r_s_	0.66	−0.72	0.67	0.41	0.36	0.77				
	*p*	<0.05	<0.05	<0.05	<0.05	<0.05	<0.05				
S_mol_	r_s_	−0.34	0.023	−0.064	0.023	0.051	−0.33	−0.33			
	*p*	NS	NS	NS	NS	NS	NS	NS			
N_mol_	r_s_	−0.051	−0.077	−0.097	0.02	0.032	−0.33	0.29	0.89		
	*p*	NS	NS	NS	NS	NS	NS	NS	<0.05		
J’	r_s_	−0.018	−0.089	−0.059	0.56	−0.69	0.015	−0.055	−0.41	−0.74	
	*p*	NS	NS	NS	NS	NS	NS	NS	<0.05	<0.05	
H’	r_s_	−0.027	−0.005	−0.51	0.031	0.008	−0.38	−0.38	0.95	0.76	−0.16
	*p*	NS	NS	NS	NS	NS	NS	NS	<0.05	<0.05	NS

## Data Availability

Raw data are available in Supplementary Materials.

## Supplementary Materials

The following supporting information can be downloaded at: https://www.mdpi.com/article/10.3390/plants11162131/s1, Supplement 1: Taxonomic list with percentage of frequency (%F), abundance (%Ab) and feeding mode (MG = micrograzers; SF = suspension feeders; DF = deposit feeders; P = predators; SP = spongivores) of each mollusc taxa. Supplement 2: PERMANOVA table of results. df = degrees of freedom; SS = sums of squares; MS = mean squares; Pseudo-F = pseudo-F ratio; res = residuals; F = F-ratio; P = permutational probability. Significant *p*-values are in bold. Supplement 3: RDA and CCA model results. Df = degrees of freedom; SS = sums of squares; F = F-ratio; P = *p*-value. Significant *p*-values are in bold.
